# Mechanisms underlying exercise intolerance in long COVID: An accumulation of multisystem dysfunction

**DOI:** 10.14814/phy2.15940

**Published:** 2024-02-12

**Authors:** Alexandra Jamieson, Lamia Al Saikhan, Lamis Alghamdi, Lee Hamill Howes, Helen Purcell, Toby Hillman, Melissa Heightman, Thomas Treibel, Michele Orini, Robert Bell, Marie Scully, Mark Hamer, Nishi Chaturvedi, Hugh Montgomery, Alun D. Hughes, Ronan Astin, Siana Jones

**Affiliations:** ^1^ MRC Unit for Lifelong Health & Ageing at UCL University College London London UK; ^2^ Department of Cardiac Technology, College of Applied Medial Sciences Imam Abdulrahman Bin Faisal University Dammam Saudi Arabia; ^3^ Department of Respiratory Medicine University College London Hospitals NHS Foundation Trust London UK; ^4^ Respiratory Medicine University College London London UK; ^5^ Barts Heart Centre, St Bartholomew's Hospital London UK; ^6^ Hatter Cardiovascular Institute University College London London UK; ^7^ Department of Haematology University College London Hospitals NHS Foundation Trust London UK; ^8^ Division of Surgery and Interventional Science University College London London UK; ^9^ Centre for Human Health and Performance University College London London UK; ^10^ National Institute for Health Research (NIHR) Biomedical Research Centre (BRC) London UK

**Keywords:** cardiopulmonary fitness, exercise intolerance, long COVID, skeletal muscle

## Abstract

The pathogenesis of exercise intolerance and persistent fatigue which can follow an infection with the SARS‐CoV‐2 virus (“long COVID”) is not fully understood. Cases were recruited from a long COVID clinic (*N* = 32; 44 ± 12 years; 10 (31%) men), and age‐/sex‐matched healthy controls (HC) (*N* = 19; 40 ± 13 years; 6 (32%) men) from University College London staff and students. We assessed exercise performance, lung and cardiac function, vascular health, skeletal muscle oxidative capacity, and autonomic nervous system (ANS) function. Key outcome measures for each physiological system were compared between groups using potential outcome means (95% confidence intervals) adjusted for potential confounders. Long COVID participant outcomes were compared to normative values. When compared to HC, cases exhibited reduced oxygen uptake efficiency slope (1847 (1679, 2016) vs. 2176 (1978, 2373) mL/min, *p* = 0.002) and anaerobic threshold (13.2 (12.2, 14.3) vs. 15.6 (14.4, 17.2) mL/kg/min, *p* < 0.001), and lower oxidative capacity, measured using near infrared spectroscopy (*τ*: 38.7 (31.9, 45.6) vs. 24.6 (19.1, 30.1) s, *p* = 0.001). In cases, ANS measures fell below normal limits in 39%. Long COVID is associated with reduced measures of exercise performance and skeletal muscle oxidative capacity in the absence of evidence of microvascular dysfunction, suggesting mitochondrial pathology. There was evidence of attendant ANS dysregulation in a significant proportion. These multisystem factors might contribute to impaired exercise tolerance in long COVID sufferers.

## 1 INTRODUCTION

Long COVID or post‐acute sequelae of SARS‐CoV‐2 infection (PASC), is defined as the presence of persistent, often severely debilitating, symptoms beyond 12 weeks from an acute episode of COVID‐19. In total, 3.1% of the UK population self‐report long COVID symptoms (Office for National Statistics, 2023) and it can occur irrespective of the severity of acute COVID‐19 symptoms. Thus, understanding the pathogenesis of long COVID symptoms is an important clinical challenge.

Extreme fatigue and exercise intolerance are common symptoms of long COVID (Greenhalgh et al., 2020; Sudre et al., 2021), many studies have reported severe reductions in exercise capacity or cardiopulmonary fitness (Contreras et al., 2023; Singh et al., 2022) however, the pathogenesis of this impairment is not fully understood. Impairments of peripheral oxygen extraction have been implicated (Contreras et al., 2023; Singh et al., 2022). One recent study performed comprehensive assessments of skeletal muscle physiology and showed a reduction in oxidative capacity and mitochondrial enzymes (Colosio et al., 2023). However, deficits across multiple physiological systems may also contribute to exercise intolerance. Dysautonomia (Dani et al., 2021; Ladlow et al., 2022; Raj et al., 2021), anemia (Baratto et al., 2021), and a range of cardiovascular abnormalities, including right ventricular dysfunction and arrhythmias, have been described in the presence of long COVID (Raman et al., 2022); all of which are known to impact exercise performance.

We aim to describe, within one study, the function of several key physiological systems important for exercise performance (cardiac, pulmonary, skeletal muscle, and ANS function) in people with symptoms of long COVID compared to healthy control participants and, for clinical context, compare them to accepted normal clinical thresholds (where available from guideline documents). Using statistical modeling we also explore whether deficits in any of these systems can explain the reduction in exercise performance in people with long COVID. We hypothesize that peripheral impairments to skeletal muscle energetics will explain deficits in aerobic capacity in long COVID.

## 2 METHODS

The study was performed in accordance with the principles of the declaration of Helsinki. All measures undertaken by participants with long COVID were reviewed by the Leicester Central Research Ethics Committee and approved by the Health Research Authority (HRA) and Health and Care Research Wales (HCRW). Ethical approval for all measurements undertaken by healthy individuals was granted by the University College London Research Ethics Committee. The study was registered on ClinicalTrials.gov (NCT04914754).

### 2.1 Study participants

Long COVID cases were identified by clinicians in the adult post COVID‐19 clinic at University College London Hospitals NHS Foundation Trust (UCH). Patients were considered eligible for the study if they had self‐reported exercise intolerance and fatigue that developed during or after an acute COVID‐19 infection (confirmed by SARS‐CoV‐2 PCR testing), which persisted for ≥12 weeks, and which was not explained by an alternative diagnosis. Healthy adult controls were recruited from University College London (UCL) staff and students and were sex‐ and age‐ (5‐year banding) matched to cases. Full exclusion criteria are defined in the Appendix S1 (Section 1.1). Individuals with long COVID performed an extended version of the study protocol to assess additional measures of cardiac, muscle, vascular and autonomic function. All research procedures took place at the Bloomsbury Centre for Clinical Phenotyping (BCCP), UCL. All participants gave written informed consent.

### 2.2 Participant characteristics and anthropometrics

Participant age, sex, ethnicity, comorbidities, and long COVID symptomatology were collected by questionnaire and recorded in REDCap. Height was measured using a stadiometer (Seca217, Seca, Germany) to the closest centimeter. Weight was measured and body fat (%) and muscle mass (%) estimated using digital bio‐impedance scales (BC‐418, Tanita, USA). Waist and hip circumference were measured using a tape measure to the closest centimeter.

### 2.3 Cardiopulmonary exercise testing (CPET)

A CPET was performed on a semi‐recumbent cycle ergometer (Ergoselect1200, Ergoline, Germany) using a ramp protocol (Wasserman et al., 2005). The test was terminated if the participant (i) reached 85% of their age‐predicted (220‐age) maximum heart rate, (ii) experienced limiting symptoms, or (iii) developed arrhythmia, hypotension (systolic blood pressure (BP) drop of >10 mmHg despite increased workload), or (iv) an excessive blood pressure rise during the test (to >250 systolic or >115 diastolic mmHg).

Expired gases were analyzed breath‐by‐breath, and heart rate and rhythm measured using a 6‐lead ECG (Quark CPET, COSMED, Italy). Peak oxygen consumption (peak V̇O_2_) was measured as the highest 30 s rolling average V̇O_2_ value during exercise. Extrapolated V̇O_2_max was calculated as peak V̇O_2_ extrapolated to age‐predicted maximum HR. Peak HR, oxygen uptake efficiency slope (OUES), anaerobic threshold (AT, if achieved), ventilatory equivalent of carbon dioxide (V̇E:V̇CO_2_), and ratio of oxygen uptake to work rate (V̇O_2_/Work Rate) were all measured. Maximum V̇O_2_ was predicted for men and women using the equations of Wasserman and Whipp (Wasserman et al., 2005). Anaerobic Threshold was determined by both ventilatory equivalent and V‐slope methods and is also presented as a percentage of predicted V̇O_2_max. Respiratory exchange ratio (RER) was calculated as V̇CO_2_/V̇O_2_.

Tests were conducted with continuous monitoring of the oxygen saturation of peripheral arterial blood (SpO_2_, finger or forehead probe) and BP was measured using a motion insensitive device (Tango M2, SunTech Medical, USA) every 2–3 min throughout exercise and recovery. Participants were asked to score both breathlessness (dyspnoea) and leg fatigue using the Borg CR10 scale on termination of exercise. Capillary lactate was measured in blood sampled from the fingertip prior to exercise and at peak effort using a point‐of‐care lactate analyzer (Nova StatStrip Xpress, Nova Biomedical, UK).

### 2.4 Lung function tests

Spirometry was performed in accordance with European Respiratory Society guidance (Quanjer et al., 2012) using an Easy‐On‐PC TrueFlow Spirometer (NDD Medical Technologies, France). Forced expiratory volume in 1 s (FEV_1_; % of predicted), forced vital capacity (FVC; % of predicted), and FEV_1_/FVC ratio are presented based on equations recommended by the European Respiratory Society and European Community of Coal and Steel. FEV_1_, FVC, and FEV_1_/FVC *Z*‐scores were calculated using the Global Lung Function Initiative reference equations (Quanjer et al., 2012).

### 2.5 Cardiovascular function

Brachial blood pressure and heart rate readings were measured in the left arm with an appropriately sized cuff using a MIT Elite Plus (Omron, The Netherlands) in a resting seated position. Clinic BP and HR were estimated as the average of the final two of three consecutive readings.

Participants with long COVID underwent a standard transthoracic echocardiogram (EPIQ 7G, Philips, MA, USA). Left ventricular (LV) structure and systolic and diastolic function were assessed using 2D, 3D, and Doppler echocardiography. Full details of the protocol and outcome measures is provided in the Appendix S1 (Section 1.2.1).

In long COVID cases, pulse wave velocity (PWV) was measured in a semi supine position using a Vicorder device (Skidmore Medical, Germany) according to manufacturer guidelines. Three consecutive measurements were acquired (within 0.5 m/s of each other) and averaged. Full protocol details are described in the Appendix S1 (Section 1.2.2).

### 2.6 Autonomic function

Heart rate recovery (HRR) was calculated at 1 and 2 min post‐exercise. The 30 s rolling average HR centred around 60 and 120 s of the recovery phase was subtracted from the peak HR measured during exercise. In long COVID cases, the change in BP and HR following a lying to standing maneuver were assessed using a brachial BP cuff (MIT Elite Plus, Omron, The Netherlands) to test for orthostatic hypotension (BP drop of >20 mmHg) and postural orthostatic tachycardia syndrome (POTS) (HR increase of >30 bpm on standing) (Freeman et al., 2011; Schatz et al., 1996). In long COVID cases, a 5‐min 12‐lead ECG was recorded at rest in the supine position to assess heart rate variability (HRV). Time domain (RMSSD, the root mean square successive NN differences) and frequency domain (LF, low frequency; HF, high frequency; LF normalized; LFHF, ratio of low frequency to high frequency) measures were derived following manufacturer algorithms (CardioPerfect HRV Module 1.6.7.1149, Welch Allyn, USA).

### 2.7 Muscle function

In long COVID cases, dominant hand‐grip strength (kPa; maximum of three measurements, with brief pauses between each) was measured with a pneumatic bulb hand dynamometer (Baseline, 3B Scientific, Germany). Concentric and eccentric strength of the left knee extensors/flexors was assessed at 60°/s for five repetitions using a HUMAC NORM isokinetic dynamometer (CSMi, USA). Participants were seated in an upright position with the lever arm pad secured proximal to the medial malleolus. The rotational axis of the knee was placed in line with the dynamometer axis of rotation, and 0° was determined as 0° knee extension. Tests were performed within a range of motion between 0 and 90° (individualized to each participant). The peak torque was defined as the highest point of the torque curve in the best repetition (Nm) and was normalized to body weight (Nm/kg) for knee extensor and flexor, respectively.

### 2.8 Skeletal muscle oxidative capacity, microvascular post‐occlusive reactive hyperaemia (PORH) and changes in tissue saturation index (TSI) with exercise

Continuous wave (CW) NIRS (Portamon, Artinis Medical Systems, The Netherlands) was used to assess skeletal muscle oxidative capacity and microvascular PORH. Full device and protocol details are provided in the Appendix S1 (Section 1.3.1).

Tissue saturation index (TSI), estimated using spatially resolved spectroscopy, was measured continuously from the gastrocnemius throughout the CPET. Greater decreases in TSI during exercise represent failure of oxygen supply to keep up with demand (Boezeman et al., 2016).

Adipose tissue thickness (ATT, average of three measures) overlying the NIRS measurement site was measured using an ultrasound device (EPIQ 7; Philips, USA) fitted with a high frequency transducer (L12‐5; Philips, USA).

#### 2.8.1 Post‐processing of near infrared spectroscopy (NIRS) data

NIRS data were analyzed in MATLAB R2022a (MathWorks Inc, USA) using custom written programs as previously described (Ryan et al., 2012), with post‐processing fully described in the Appendix S1 (Section 1.3.2).

### 2.9 Physical activity

#### 2.9.1 Self‐report

Participants were asked to complete the Recent Physical Activity Questionnaire (RPAQ), which assesses physical activity in four domains (leisure, work, commuting, and home) during the past month (Besson et al., 2010). Summary variables including the time (min/day) spent sedentary, and in light, moderate and vigorous activities, were derived using the MRC RPAQ data processing guidelines (MRC Epidemiology Unit, 2023).

#### 2.9.2 Wrist‐worn actigraphy

Participants were fitted with a wrist‐worn actigraphy monitor (Actiwatch Spectrum Plus, Philips, The Netherlands) on their nondominant wrist for 7 days following their clinic visit. Activity counts from the device over each 30 s epoch were used to determine sedentary, sleep and wake intervals, and derive average activity counts per minute during the day.

### 2.10 Hemoglobin status

In long COVID cases, capillary hemoglobin (g/L) was measured in blood sampled from the fingertip using a point‐of‐care analyzer prior to exercise (Hemoglobin Hb 801, HemoCue, Sweden).

### 2.11 Sample size

Sample size calculations were performed using GPower 3.1.9.7 (*α* = 0.05 (two‐tailed) and 80% power) assuming that the minimum clinically important difference for the primary outcomes (CPET, lung, muscle, and vascular function) corresponded to an effect size of 0.9 SD (representing a 10%–20% difference in outcome measure) based on previous studies (Jones et al., 2014; Jones, Tillin, et al., 2020). We aimed to recruit cases and controls in an approximate proportion of 2:1 to enhance the power of the planned sub‐study analysis (described in full on ClinicalTrials.gov NCT04914754). On this basis, we calculated that 32 and 16 participants would be required in the long COVID and control group, respectively (48 in total).

### 2.12 Statistical methods

Statistical analysis was performed in STATA 17.0 (StataCorp LLC, USA). Categorical participant data are presented as frequency (%) and continuous data presented as mean ± SD if normally distributed or median [interquartile range; IQR] if skewed. For simple comparisons of two groups a Pearson's Chi^2^ test was used to compare categorical variables and an unpaired Student's *t* test (normally distributed), or Wilcoxon rank‐sum (skewed distribution) were used for continuous variables.

Outcome measures were compared between long COVID and HC participants using causal inference methods to calculate differences and potential outcome means (POMs). This method aims to estimate the differences between groups while controlling for confounding bias present in observational studies. POMs were estimated using an augmented inverse probability weighted (AIPW) estimator with linear outcome and logit treatment models. AIPW is a statistical approach that combines propensity‐based inverse probability weighting (where the contribution of an individual's data is weighted by the propensity score) and regression adjustment. This approach has similarities to binary 1:1 matching, but is advantageous in that the entire sample is used and statistical power preserved. Additionally, AIPW is considered “doubly robust,” in that only one of the inverse probability weighting or regression adjustment need be correctly specified to obtain an unbiased effect estimator.

Estimates were adjusted for potential confounders chosen a priori (age, sex, ethnicity, and BMI) informed by a directed acyclic graph and are presented as POM (95% confidence intervals) and differences between groups for model 1 (M1). Regression diagnostics were performed, and histograms of propensity scores examined for appropriate overlapping. Missing data were dealt with via listwise deletion, which is valid under the assumption of missing completely at random.

Where well‐accepted normal ranges exist, additional measures of cardiac, muscle, vascular, and autonomic function were only performed in long COVID cases and were compared to reference normative ranges or cutoffs for normal values. Confidence intervals for the *n* (%) abnormal were estimated using the Wilson method, and *p*‐values calculated using binomial probability testing (Brown et al., 2001).

As an exploratory analysis, functional CPET outcomes were additionally adjusted for possible mediators: skeletal muscle function (muscle oxidative capacity; Model 2 (M2)), lung function (FEV_1_ *Z*‐score; Model 3 (M3)), autonomic function (HRR 2‐min; Model 4 (M4)) and combined (M2 + M3 + M4; Model 5 (M5)), to establish if differences in one or all of these domains could explain some of the differences observed in performance at CPET. The natural indirect effect (NIE) refers to the difference between no mediator in the model and the effect after the mediator has been controlled by regression. The 95% confidence intervals and *p*‐values for each NIE were calculated using bootstrapping with 1000 samples and the Wald test, respectively.

The level of significance was set at *p* < 0.05. No adjustment was made for multiple testing, and inference was made based on effect size, statistical significance and 95% confidence intervals.

## 3 RESULTS

### 3.1 Participant characteristics

**FIGURE 1:**
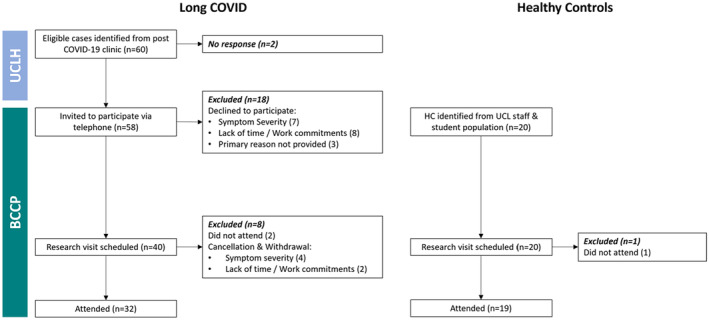
Study participant recruitment flowchart. Participants with Long COVID with self‐reported exercise intolerance and fatigue were identified from the post COVID‐19 clinic at University College London Hospital (UCLH). The study research team at Bloomsbury Centre for Clinical Phenotyping (BCCP) invited eligible individuals to attend a research visit. Healthy Control participants sex and age (5‐year banding) were recruited from the staff and student population at UCL.

In total, 32 participants with long COVID (10 (31%) men, 44 ± 12 years old) and 19 healthy controls (6 (32%) men, 40 ± 13 years old) were recruited (Figure 1). Cases had higher BMI, waist‐to‐hip ratio, body fat (%), and calf adipose tissue thickness than controls (Table 1). Among cases, 26 (81%) of participants self‐reported a preexisting condition including: hypertension, asthma, Type 2 diabetes mellitus, and mental health conditions (Table S1).

**TABLE 1:** Characteristics for 32 long COVID cases versus 19 healthy controls.

	Mean ± SD, median [IQR] or *n* (%)		
	Healthy controls (*n* = 19)	Long COVID (*n* = 32)	*p*‐value
Age [years]	40 ± 13	44 ± 12	0.235
Male sex	6 (32%)	10 (31%)	0.981
Ethnicity			
White European	15 (79%)	20 (63%)	
Indian Asian	3 (16%)	7 (22%)	0.360
Black African Caribbean	0 (0%)	4 (12%)	
Mixed	1 (5%)	1 (3%)	
BMI [kg/m^2^]	24.9 ± 3.7	28.4 ± 5.8	0.022
Waist‐to‐hip ratio	0.82 [0.09]	0.88 [0.10]	0.029
Clinic SBP [mmHg]	119 ± 14	124 ± 19	0.397
Clinic DBP [mmHg]	79 ± 11	82 ± 13	0.495
Resting HR [bpm]	73 ± 12	74 ± 13	0.877
Current smoker	0 (0%)	2 (6%)	0.270
No. of comorbidities	0	2 ± 2	<0.001
No. of long COVID symptoms	0	10 ± 4	<0.001
Body fat [%]	28 ± 7 (*n* = 18)	34 ± 7 (*n* = 28)	0.008
Estimated muscle mass [%]	69 ± 7 (*n* = 18)	62 ± 7 (*n* = 28)	0.002
Calf ATT [cm]	0.61 ± 0.16 (*n* = 17)	0.83 ± 0.34 (*n* = 25)	0.016

Abbreviations: ATT, Adipose tissue thickness; BMI, body mass index; HR, heart rate; SBP & DBP, resting systolic and diastolic blood pressure.

#### 3.1.1 Acute SARS‐CoV‐2 severity and treatment

Thirty cases had been managed at home during their acute SARS‐CoV‐2 infection, and two had required hospitalization, one received dexamethasone and tocilizumab, and the other received dexamethasone and remdesivir.

#### 3.1.2 Long COVID symptomatology

Cases were investigated in our study, on average, 14 ± 6 months post‐acute SARS‐CoV‐2 infection. On average participants with long COVID reported 10 ± 4 symptoms, and all were experiencing fatigue and exercise intolerance (Figure S1).

### 3.2 Long COVID versus healthy control participants and normative values

#### 3.2.1 Exercise performance and cardiopulmonary fitness

Four participants with long COVID had a contraindication to exercise testing on the day of their study visit and were excluded. A further two participants with long COVID were unable to exercise beyond the warm‐up phase and were excluded from subsequent analyses. Only 8 (29%) cases achieved 85% of their age‐predicted maximum heart rate compared with 14 (74%) of controls (*p* = 0.001). Of the 20 remaining cases, 15 experienced limiting symptoms, 4 had a hypotensive response (systolic BP drop of >10 mmHg with increased workload) in the absence of ischaemic ECG changes, and 1 had an exaggerated exertional blood pressure response (>250/115 mmHg).

After adjustment for age, sex, ethnicity, and BMI, cases achieved a lower exertional V̇O_2_peak and had lower extrapolated V̇O_2_max than controls (Table 2). OUES, anaerobic threshold, peak HR, O_2_ pulse, and ratio of oxygen uptake to work rate were also lower, and VE/V̇CO_2_ slope higher (Table 2). A respiratory exchange ratio (RER) of ≥1.1 was measured in 22 (85%) of cases and 17 (90%) HC. Borg CR10 scores were lower in HC than cases (5 ± 1 vs. 6 ± 2 for breathlessness and 6 ± 3 vs. 7 ± 2 for leg fatigue). Sensitivity analysis directly comparing 18 HC and 18 cases, unadjusted for confounders, showed similar trends (Table S2).

**TABLE 2:** Cardiopulmonary exercise test confounder adjusted (age, sex, ethnicity, & BMI) results for healthy controls versus long COVID cases.

Cardiopulmonary exercise test	*n*	Potential outcome means (95% CI)		The difference in long COVID versus HC	
		Adj. for age, sex, ethnicity & BMI		M1: Adj. for age, sex, ethnicity & BMI	
		Healthy controls	Long COVID	Δ (95% CI)	*p*‐value
Peak V̇O_2_ [mL/kg/min] during exercise	45	23.3 (21.4, 25.2)	18.3 (16.6, 19.7)	−5.0 (−7.1, −3.0)	<0.001
Extrapolated V̇O_2_ max [mL/kg/min]	45	33.0 (30.7, 35.3)	30.5 (28.1, 32.8)	−2.5 (−5.2, 0.2)	0.064
OUES [mL/min]	45	2175.8 (1978.3, 2373.3)	1847.4 (1678.5, 2016.4)	−328.3 (−534.4, −122.3)	0.002
V̇O_2_ at AT [mL/kg/min]	44	15.6 (14.4, 17.2)	13.2 (12.2, 14.3)	−2.4 (−3.7, −1.1)	<0.001
VAT [% of predicted]	44	56.7 (53.3, 60.0)	47.8 (44.1, 51.6)	−8.8 (−13.3, −4.4)	<0.001
V̇E/V̇CO_2_ slope	45	25.7 (24.5, 26.8)	28.0 (26.9, 29.2)	2.4 (0.9, 3.8)	0.001
Peak HR [min^−1^]	45	146 (143, 149)	130 (119, 140)	−16 (−27, −5)	0.003
Peak HR [% predicted]	45	82 (81, 84)	73 (68, 79)	−9 (−15, −3)	0.002
Highest V̇O_2_/HR (O_2_ pulse) [mL/beat]	45	12.0 (10.8, 13.1)	10.5 (9.6, 11.3)	−1.5 (−2.5, −0.5)	0.002
V̇O_2_/WR [mL/min/W]	45	9.3 (8.9, 9.8)	8.7 (8.2, 9.1)	−0.7 (−1.3, −0.1)	0.027
Peak power [W]	45	170.5 (154.5, 186.4)	128.2 (112.9, 143.4)	−42.3 (−56.6, −28.0)	<0.001
Peak ventilation [L/min]	45	58 (52, 64)	48 (41, 54)	−11 (−18, −4)	0.001
Peak breathing reserve [%]	45	57 (52, 63)	59 (53, 66)	2 (−6, 11)	0.607
Peak RER	45	1.18 (1.15, 1.21)	1.11 (1.06, 1.17)	−0.07 (−0.13, −0.01)	0.024
No. of participants RER ≥1.1		22 (85%)	17 (90%)	‐	‐
Lactate [Δmmol/L]	36	4.5 (3.5, 5.5)	2.6 (1.5, 3.7)	−1.9 (−3.2, −0.6)	0.003
Borg CR10 dyspnoea	44	5 (5, 6)	6 (5, 6)	0 (−1, 1)	0.500
Borg CR10 leg fatigue	45	6 (4, 7)	7 (6, 8)	1 (−0, 3)	0.139

Abbreviations: AT, Anaerobic threshold; OUES, oxygen uptake efficiency slope; RER, respiratory exchange ratio; V̇E/V̇CO_2_, ventilation/carbon dioxide production; V̇O_2_, oxygen uptake; V̇O_2_WR, oxygen uptake work rate.

Of the participants with long COVID, 32% fell below the normal cutoff for extrapolated V̇O_2_max, 31% for OUES and 46% for V̇O_2_/WR. About 14% met criteria for V̇E/V̇CO_2_ abnormality (slope >30), but we did not observe any individuals who were below normal limits for breathing reserve (<15 L).

#### 3.2.2 Lung function

FEV1 and FVC (percentage of predicted and *Z*‐scores) were lower in cases than controls (Table 4). However, means all fell within normal ranges (above LLN) and 69% had normal spirometry as defined by FEV_1_, FVC, and FEV_1_/FVC *Z*‐score >−1.64.

#### 3.2.3 Cardiac structure and function

We did not find strong evidence of systolic or diastolic cardiac dysfunction in cases assessed at rest by echocardiography. The prevalence of abnormal LV relaxation (e′) in 19%–25% of long COVID individuals was higher than anticipated, although none met clinical criteria defining diastolic dysfunction. All participants fell within normal limits for LVEF measured by 3D echocardiography, a method that avoids geometric assumptions (Table 3).

**TABLE 3:** Additional measures of cardiac, vascular, muscle and autonomic function and hemoglobin status for participants with long COVID.

	Additional measures in participants with long COVID				
	Mean ± SD median [IQR] or *n* (%)	Normal range or cut‐off for normal	Reference	Participants outside of normative values *n* [% (95% CI)]	*p*‐value
Cardiopulmonary exercise test	(*n* = 28)				
Extrapolated V̇O_2_ max [mL/kg/min]	30 ± 7	^a^	Wasserman et al. (2005)	9 [32% (18, 51%)]	<0.001
OUES [mL/min]	1877 ± 495	<1500	Wasserman et al. (2005)	8 [31% (15, 47%)]	<0.001
V̇E/V̇CO_2_ slope	28 ± 3	>30	Nayor et al. (2020)	4 [14% (6, 32%)]	0.049
V̇O_2_/WR [mL/min/W]	8.6 ± 1.4	<8.5	Wasserman et al. (1999)	13 [46% (30, 64%)]	<0.001
Breathing reserve [L/min]	72 ± 27	<15	American Thoracic Society and American College of Chest Physicians (ACCP) (2003)	0 [0%]	‐
Lung function	(*n* = 32)				
Abnormal spirometry	‐	<−1.64	Haynes (2018)	10 [31% (18, 49%)]	<0.001
Restrictive spirometry pattern	‐	FVC < −1.64 & FEV_1_/FVC > −1.64	Haynes (2018)	5 [16% (7, 32%)]	0.020
Obstructive spirometry pattern	‐	FEV_1_/FVC < −1.64	Haynes (2018)	2 [6% (2, 20%)]	0.480
Reduced FEV_1_ only	‐	FEV_1_ < −1.64 & FVC > −1.64 & FEV_1_/FVC > −1.64	Haynes (2018)	3 [9% (3, 24%)]	0.214
Echocardiography	(*n* = 32)				
Systolic function					
3D LVEF [%]	66 ± 8	>55	Lang et al. (2015)	0 [0%]	‐
3D GLS [%]	−24.6 [4.2]	<−14	Lang et al. (2015)	0 [0%]	‐
Diastolic function					
Average E/e′	7.5 [2.6]	≤14	Nagueh et al. (2016)	1 [3% (1, 16%)]	0.806
Septal e′ velocity [cm/s]	9.0 [2.4]	≥7	Nagueh et al. (2016)	8 [25% (13, 42%)]	<0.001
Lateral e′ velocity [cm/s]	12.2 [3.4]	≥10	Nagueh et al. (2016)	6 [19% (9, 35%)]	0.005
TR velocity [m/s]	NA	≤2.8	Nagueh et al. (2016)	0 [0%]	‐
LA volume index [mL/m^2^]	21.1 [5.3]	≤34	Nagueh et al. (2016)	0 [0%]	‐
Presence of diastolic dysfunction by ASE criteria	0 (0%)	>50% indicators positive	Nagueh et al. (2016)	0 [0%]	‐
Vascular function	(*n* = 32)				
Peripheral SBP	122 [21]	≤140	Jones, McCormack, et al. (2020)	4 [13% (5, 28%)]	0.074
Peripheral DBP	82 [14]	≤90	Jones, McCormack, et al. (2020)	8 [25% (13, 42%)]	<0.001
Peripheral HTN (either SBP or DBP HTN)	(*n* = 24)	≥140/90	Jones, McCormack, et al. (2020)	9 [28% (14, 47%)]	<0.001
Pulse wave velocity [m/s]	7.5 ± 1.2	^b^	Reference Values for Arterial Stiffness' Collaboration (2010)	0 [0%]	‐
Muscle function	(*n* = 27)				
Hand‐grip strength [kPa]	71.9 ± 18.3	^b^	Massy‐Westropp et al. (2011)	2 [7% (2, 23%)]	0.394
	(*n* = 13)				
Knee extensor Pk. torque [Nm/kg]	1.45 ± 0.67	Female: 1.26 (1.01–1.50) Male: 1.79 (1.34–2.23)	Šarabon et al. (2021)	2 [15% (4, 42%)]	0.135
Knee flexor Pk. torque [Nm/kg]	0.71 ± 0.36	Female: 1.00 (0.78–1.21) Male: 1.03 (0.85–1.20)	Šarabon et al. (2021)	8 [62% (36, 82%)]	<0.001
Autonomic function	(*n* = 28)				
SBP↓ after 1 min standing [mmHg]	1 ± 11				
SBP↓ after 3 min standing [mmHg]	−2 ± 11	≤20	Schatz et al. (1996)	1 [4% (1, 18%)]	0.762
HR↑ after 1 min standing [bpm]	14 ± 9				
HR↑ after 3 min standing [bpm]	12 ± 8	≤30	Freeman et al. (2011)	2 [7% (2, 23%)]	0.412
Heart rate variability	(*n* = 26)				
RMSSD [ms]	29 [29]	≥19	Nunan et al. (2010)	5 [19% (9, 38%)]	0.009
LF [ms^2^]	344 [734]	≥193	Nunan et al. (2010)	10 [39% (22, 57%)]	<0.001
LF normalized	49 [35]	≥30	Nunan et al. (2010)	4 [15% (6, 34%)]	0.039
HF [ms^2^]	254 [828]	≥83	Nunan et al. (2010)	5 [19% (9, 38%)]	0.009
LFHF	0.95 [1.54]	≤11.6	Nunan et al. (2010)	0 [0%]	‐
↓RMSSD, ↓HF and ↓LF & ↑LF/HF ratio	‐	‐	‐	0 [0%]	‐
Presence of at least one abnormal autonomic parameter	(*n* = 28)	‐	‐	10 [36% (21, 54%)]	<0.001
Hemoglobin status	(*n* = 25)				
Hemoglobin (Hb) [g/L]	135 ± 11	Female: >120 g/L Male: >130 g/L	World Health Organization (2011)	5 [20% (9, 39%)]	0.007

*Note*: Details are provided in full in references provided.

Abbreviations: ASE, American Society of Echocardiography; DBP, diastolic blood pressure; FEV_1_, forced expiratory volume in the 1st second; FEV_1_/FVC ratio, ratio of the forced expiratory volume in the 1st second to the forced vital capacity; FVC, forced vital capacity; GLS, global longitudinal strain; HF, high frequency; HR, heart rate; LA, left atria; LF, low frequency; LFHF, low frequency high frequency ratio; LVEF, left ventricular ejection fraction; OUES, oxygen uptake efficiency slope; Pk, Peak; RMSSD, root mean square of successive differences between normal beats; SBP, systolic blood pressure; TR, tricuspid regurgitation; V̇E/V̇CO_2_, ventilation/carbon dioxide production; V̇O_2_, oxygen uptake; V̇O_2_WR, oxygen uptake work rate.

^a^ Normative values were calculated using sex specific equations.

^b^ Normative values were stratified by sex and age decade.

#### 3.2.4 Skeletal muscle function

A total of six NIRS recordings were rejected: five due to apparent failure of arterial occlusion, and one due to the participant not fully recovering from the exercise after 3‐min of transient occlusions (data did not fit to the mono‐exponential curve and plateau not achieved). Resting muscle oxygen consumption (musV̇O_2_) was similar but the time constant for the recovery of muscle V̇O_2_ was longer in cases than controls (Table 4). Complete arterial occlusions for the PORH measure were on average 240 ± 60 s. There were no differences in time to 95% peak hyperemia and change in TSI during exercise (Table 4). Hand‐grip strength was 71.9 ± 18.3 kPa and knee extensor/flexor peak torque was 1.45 ± 0.67 and 0.71 ± 0.36 Nm/kg, respectively (Table 3). Muscle strength measures were below normal limits in 7% of cases for hand‐grip strength, 15% for knee extensor, and 62% for knee flexor.

**TABLE 4:** Lung function (spirometry), Vascular function (resting blood pressure), skeletal muscle function (near infrared spectroscopy), microvascular function (near infrared spectroscopy), autonomic function (heart rate recovery) and physical activity (self‐reported and actigraphy) confounder adjusted (age, sex, ethnicity, & BMI) results for healthy control participants versus long COVID cases.

	*n*	Potential outcome means (95% CI)		The difference in long COVID versus HC	
		Adj. for age, sex, ethnicity & BMI		M1: Adj. for age, sex, ethnicity & BMI	
		Healthy controls	Long COVID	Δ (95% CI)	*p*‐value
Lung function					
FEV_1_ pred. [%]	51	109 (103, 115)	94 (89, 99)	−15 (−23, −7)	<0.001
FVC pred. [%]	51	114 (105, 122)	96 (91, 101)	−18 (−27, −8)	<0.001
FEV_1_/FVC	51	0.82 (0.79, 0.84)	0.83 (0.81, 0.85)	0.01 (−0.02, 0.05)	0.410
FEV_1_ *Z*‐score	51	0.2 (−0.2, 0.7)	−0.8 (−1.2, −0.4)	−1.0 (−1.7, −0.4)	0.001
FVC *Z*‐score	51	1.2 (0.2, 2.1)	−0.9 (−1.3, −0.6)	−2.1 (−3.2, −1.1)	<0.001
FEV_1_/FVC *Z*‐score	51	−0.7 (−1.3, −0.1)	0.2 (−0.2, 0.5)	0.8 (0.1, 1.5)	0.018
Vascular function					
Peripheral SBP	51	121 (116, 126)	122 (117, 127)	2 (−6, 7)	0.668
Peripheral DBP	51	81 (77, 85)	81 (77, 85)	0 (−5, 5)	0.956
Skeletal muscle microvascular function					
Δ TSI on exercise	39	4.3 (2.6, 6.0)	5.2 (3.8, 6.6)	0.9 (−1.3, 3.1)	0.410
Time to 95% Pk hyperaemia [s]	27	28.0 (23.7, 32.3)	27.2 (22.0, 32.5)	−0.8 (−7.9, 6.4)	0.829
Skeletal muscle oxidative capacity					
Resting mV̇O_2_ [ΔHbdiff μM/s]	38	0.15 (0.12, 0.18)	0.12 (0.08, 0.15)	−0.03 (−0.08, 0.01)	0.110
Time constant *τ* [s]	36	24.2 (18.4, 30.0)	39.3 (32.3, 46.2)	15.1 (6.0, 24.1)	0.001
Autonomic function					
HRR at 1‐min [bpm]	44	36 (32, 41)	31 (28, 35)	−5 (−11, 1)	0.125
HRR at 2‐min [bpm]	44	50 (44, 55)	43 (38, 48)	−7 (−14, 1)	0.078
Physical activity					
Self‐reported (RPAQ)					
Time spent sedentary, excluding sleep [min/day]	47	538 (461, 614)	466 (387, 546)	−72 (−182, 39)	0.203
Time spent in LPA light intensity activity [min/day]	47	35 (−16, 87)	72 (30, 114)	37 (−29, 103)	0.270
Time spent in MPA [min/day]	47	37 (22, 52)	42 (6, 77)	4 (−35, 42)	0.836
Time spent in VPA [min/day]	47	4 (2, 6)	2 (−1, 4)	−3 (−6, 0)	0.037
Actigraphy					
Time spent in sedentary, excluding sleep [min/day]	47	52 (42, 62)	66 (58, 75)	14 (2, 27)	0.025
Time spent in activity [min/day]	47	658 (611, 704)	612 (568, 656)	−45 (−107, 16)	0.146
Average activity counts during the day [counts per minute]	47	125 (112, 137)	116 (98, 134)	−8 (−31, 15)	0.473
Time spent in sleep [min/day]	47	394 (359, 429)	438 (412, 464)	44 (7, 82)	0.020

Abbreviations: DBP, Diastolic blood pressure; FEV_1_, forced expiratory volume in the 1st second; FEV_1_/FVC ratio, FVC, forced vital capacity; HRR, heart rate recovery; LPA, light physical activity; MPA, moderate physical activity; mV̇O_2_, muscle oxygen uptake; RPAQ, Recent Physical Activity Questionnaire; SBP, systolic blood pressure; TSI, tissue saturation index; VPA, vigorous physical activity.

#### 3.2.5 Autonomic function

Despite achieving a lower peak HR, HR change after 1 min of recovery was similar between cases and controls. However, HR change after 2 min of recovery was less in cases (Table 4). One individual met the criteria for orthostatic hypotension and 2 (7%) individuals met the criteria for POTS. At least one resting HRV parameter fell outside normative ranges in 39% of cases (Table 3). Additional HRV parameters are summarized in the Appendix S1 (Table S3).

#### 3.2.6 Physical activity (PA)

Differences were not observed between groups in self‐reported RPAQ time spent in sedentary, light and moderate PA. However, cases reported spending less time in vigorous activity than controls (Table 4). PA assessed via actigraphy showed more time sedentary (66 (58, 75) vs. 52 (42, 62) min, *p* = 0.025) and sleeping (438 (412, 464) vs. 394 (359, 429) min, *p* = 0.020) in cases than controls. However, differences were not observed in the time spent in activity (612 (568, 656) vs. 658 (611, 704) min, *p* = 0.473) and average number of activity counts per minute (116 (98, 134) vs. 125 (112, 137) min, *p* = 0.146) between groups (Table 4).

#### 3.2.7 Hemoglobin status

5 (20%) cases fell below the normal cutoffs for hemoglobin levels (Table 3), three of which were included in CPET analyses.

### 3.3 Mediation analysis

**FIGURE 2:**
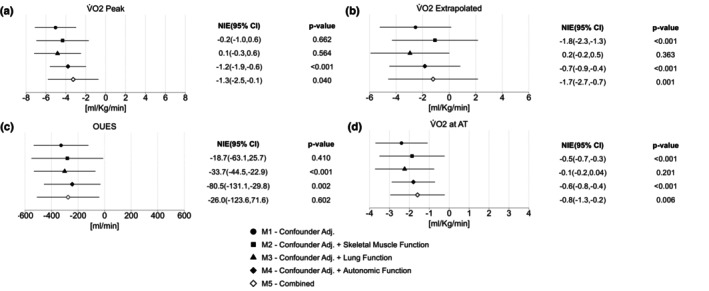
Forest plots for the mediation analysis of cardiopulmonary exercise test outcomes: (a) V̇O_2_ peak, (b) V̇O_2_ extrapolated, (c) OUES and (d) V̇O_2_ at AT. The natural indirect effect (NIE) refers to the difference between no mediator in the model and the effect after the mediator has been controlled by regression. The 95% confidence intervals and *p*‐values relate to each NIE. Model 1 (M1): confounder adjusted for age, sex, ethnicity and body mass index (BMI); Model 2 (M2): confounder adjusted + adjusted for skeletal muscle function (time constant); Model 3 (M3): confounder adjusted + adjusted for lung function (FEV_1_ *Z*‐score); Model 4 (M4): confounder adjusted + adjusted for autonomic function (heart rate recovery (HRR) at 2‐min); and Model 5 (M5): confounder adjusted + adjusted for time constant, FEV_1_ *Z*‐score and HRR at 2‐min (combined). AT, Anaerobic threshold; OUES, oxygen uptake efficiency slope; V̇O_2_, oxygen uptake.

To explore whether differences in skeletal muscle, lung, autonomic, and cardiac function (separately and combined) might explain the differences in exercise performance observed between cases and controls, we performed an exploratory analysis in which we further adjusted our models for the following: time constant (M2), FEV_1_ *Z*‐score (M3), HRR 2‐min (M4), and M2 + M3 + M4 combined (M5). Model 1–5 estimations (95% confidence intervals) for key CPET outcomes are illustrated as forest plots in Figure 2.

The differences in extrapolated V̇O_2_max between cases and controls were attenuated from −2.9 to −1.1 (*p* < 0.001) by the time constant (M2) and −2.5 to −1.8 (*p* < 0.001) by HRR at 2 min (M4). Evidence of partial mediation was observed in each of the investigated systems: for the time constant (M2), this included V̇O_2_ at AT; for FEV_1_ *Z*‐score (M3), this included OUES and V̇O_2_ at AT and for HRR at 2 min (M4), this included OUES.

## 4 DISCUSSION

Individuals experiencing symptoms of COVID beyond 12 weeks post‐infection have reduced skeletal muscle oxidative capacity, lung function and ANS function compared to age, sex, and BMI‐matched healthy control participants. Compared to normal clinical ranges, ~30% of participants with long COVID fell outside the normal range for spirometry and 36% for ANS function. However, CPET indices suggest exercise performance was limited by peripheral factors, rather than lung or circulatory dysfunction. Through exploratory mediation analyses, we did not find strong evidence that any one physiological deficit fully explained the severely impaired exercise performance in long COVID; this points to a multisystem dysfunction contributing to exercise impairment. It is important to recognize that our study sample was small for mediation analysis and we interpret these results with caution.

The observed reduction in exercise performance in long COVID is aligned with prior studies (Alba et al., 2021; Barbagelata et al., 2022; Brown et al., 2022; Contreras et al., 2023; de Boer et al., 2022; Debeaumont et al., 2021; Dorelli et al., 2021; Frésard et al., 2022; Ladlow et al., 2022; Mancini et al., 2021; Mohr et al., 2021; Norweg et al., 2023; Singh et al., 2022). We present maximal and sub‐maximal indices of cardiopulmonary fitness, all indicating impairments in performance and cardiopulmonary fitness. O_2_ pulse was lower in cases than controls in line with prior findings (Cassar et al., 2021; Miętkiewska‐Szwacka et al., 2023). A low O_2_ pulse can indicate impaired augmentation of stroke volume or impaired peripheral oxygen extraction during exercise. The reduced AT and V̇O_2_/WR ratio in long COVID cases point toward a peripheral limitation. This is in line with findings by Singh et al. (2022) who reported that exercise capacity beyond 75% of peak V̇O_2_ was limited by a failure to increase peripheral oxygen extraction.

Our finding that individuals with long COVID had poorer skeletal muscle oxidative capacity further supports a peripheral skeletal muscle impairment in the presence of long COVID. This is concordant with a study by Colosio et al. (2023) who performed high‐resolution respirometry on skeletal muscle biopsies and noninvasive NIRS measurements, similar to our NIRS measurements, in people with long COVID and healthy controls. Together, these findings provide evidence for a deficit in skeletal muscle mitochondrial bioenergetics in long COVID. Our larger sample size, compared to Colosio et al. (2023), allowed us to perform additional statistical analyses exploring the role of oxidative capacity in mediating the effect of long COVID on exercise performance. When we included oxidative capacity (*τ*) as a mediator in statistical models, we observed that the differences observed in extrapolated V̇O_2_max and V̇O_2_ at AT between cases and controls were partially attenuated. Partial mediation was also observed when we included the ANS system and lung function measures into the model, therefore, we cannot exclude the possibility of multisystem involvement in the exercise performance impairment observed in long COVID participants. Our study sample size is small for this type of analysis which limits our power to perform multivariable adjustments, and this could have impacted our results. Larger studies are necessary to confirm these effects.

Support for a skeletal muscle deficit more generally was also evidenced by the reduced knee extension/flexion force in 62% of long COVID cases enrolled here. This may be explained by the reduced lean mass in our study group, as was described by Ramirez‐Velez and colleagues (Ramírez‐Vélez et al., 2023). Only 7% of individuals with long COVID had a hand‐grip strength outside the normal range (Ramírez‐Vélez et al., 2023), which may suggest a detraining effect on the lower limbs in line with the increase in sedentary time that we observed objectively. However, we also report similar self‐reported and objectively measured levels of physical activity between groups which would argue against detraining. A major limitation of this study, and many other long COVID studies, is that we cannot be certain whether the deficits described existed prior to COVID‐19 infection or are the result of infection. Studies where antecedent measurements have been performed would be extremely useful in understanding the development of pathophysiology in long COVID.

There was no strong evidence of resting cardiac structural or functional abnormalities or evidence of increased stiffening of the arterial system in our long COVID cases. Time to 95% PORH measured using NIRS was similar in cases and controls. This suggests reactivity in the microcirculation, an indicator of endothelial function, is not impaired in long COVID (Boezeman et al., 2016). In four long COVID participants resting blood pressure was above the cutoff for both systolic and diastolic hypertension (>140/90 mmHg). All four participants reported a diagnosis of hypertension prior to COVID‐19 infection and were being treated with antihypertensive agents, none were treated with a β‐blocker.

### 4.1 Autonomic function

There was heterogeneity across measures of autonomic function in long COVID cases. On average, HR recovered more rapidly in controls than in long COVID cases, in line with previous work (Asarcikli et al., 2022; Contreras et al., 2023; Dani et al., 2021; Marques et al., 2022; Suh et al., 2023). During the lying to standing maneuver, one individual met the clinical criteria for orthostatic hypotension and a further two met the criteria for POTS (Raj et al., 2021). We also observed exaggerated heart rate increases on standing (>20 bpm) in a further seven individuals, suggesting possible subclinical abnormalities. A limitation is that HR and BP measurements were not continuous, meaning that we were only able to assess single measurement differences, increasing the possibility of measurement errors. No long COVID participants fell outside of normal limits for all three key HRV variables measured (LF, HF, and RMSSD); however, 39% fell below normal limits for the LF variable and 18% below normal limits for the RMSSD and HF variables, a finding in line with HRV alterations previously described in long COVID case–control studies (Kurtoğlu et al., 2022; Mooren et al., 2023).

### 4.2 Strengths and limitations

Causal inferences are limited in our study by small sample size and cross‐sectional design. We cannot be certain whether observed pathophysiology in long COVID participants was the cause or consequence of their symptoms, or secondary to their preexisting (non‐COVID) states. Likewise, we cannot confirm whether differences in body composition were due to long COVID‐related inactivity or a secondary (or preexisting) low‐grade metabolic syndrome that would predispose to poorer oxidative capacity in muscle. We performed additional measures of cardiac, autonomic, vascular, and skeletal muscle function in participants with long COVID only and used reference normative values to identify those who fell outside of normal limits. However, we cannot rule out the fact that these subclinical values might not have also been observed in the general population at the same frequency. We used NIRS to measure oxidative capacity, and the PORH response to ischaemia as a measure of microvascular function (Rosenberry & Nelson, 2020). There are some limitations to NIRS, discussed in detail elsewhere (Jones et al., 2016). The gold‐standard noninvasive method for assessing oxidative capacity is to directly measure PCr recovery using ^31^P‐MRS, confirmation of these findings via this method would be useful.

One limitation is that CPET was terminated at 85% of predicted maximum HR. This exercise protocol was established in consideration of safety concerns during the early stages of the pandemic when the study was designed and in accordance with the restrictions imposed by the research ethics committee. Exercise was limited by symptoms in 58% of long COVID cases providing a measured maximal V̇O_2_ in those individuals, and demonstrated reduced cardiopulmonary fitness. Furthermore, a RER of ≥1.1 was measured in 85% of cases and 90% of controls indicating that the majority of individuals were nearing peak effort.

## 5 CONCLUSIONS

We have identified a limitation in peripheral oxygen uptake with normal local vascular supply in the presence of long COVID, suggesting pathophysiology of mitochondrial oxygen uptake and utilization. Understanding the exact mechanism of the myocellular defect is important to identify therapeutic targets. We also observed some evidence for lung and autonomic dysfunction highlighting that exercise intolerance in long COVID may not be attributed to impairment in a single physiological system, but rather the result of an accumulation of multisystem, often subclinical, dysfunction, and their interplay.

## AUTHOR CONTRIBUTIONS

All authors contributed to study design, data interpretation and revision of the manuscript. Participant recruitment was performed by Ronan Astin, Melissa Heightman, Toby Hillman, Helen Purcell, Alexandra Jamieson and Siana Jones. Data capture was performed by Alexandra Jamieson, Siana Jones and Lamia Al Saikhan. Data processing and statistical analyses was performed by Alexandra Jamieson with support from Siana Jones, Ronan Astin, Alun Hughes and Michele Orini. Echocardiography data was processed by Lamis Alghamdi. Hugh Montgomery, Thomas Treibel, Robert Bell, Marie Scully, Mark Hamer, Nishi Chaturvedi and Lee Hamill Howes were involved in the conceptualisation of the study, study design, investigation and reviewing all methods and manuscripts. All authors have approved the final version of the manuscript. We are a multi‐disciplinary team that has greatly benefited from the expertise of the authors who specialize in each of the physiological systems investigated in this study.

## FUNDING INFORMATION

The study was undertaken as part of a 4‐year PhD studentship supported by the British Heart Foundation (grant number FS/19/63/34902). NC and ADH work in a unit that receives support from the UK Medical Research Council (grant number MC_UU_12019/1). HM is supported by the National Institute for Health Research's Comprehensive Biomedical Research Centre at University College Hospital London.

## CONFLICT OF INTEREST STATEMENT

The authors declare no conflicts of interest.

## Supporting information


Appendix S1

## Data Availability

Due to the sensitive nature of the data collected for this study, data cannot be made publicly available, but requests to access the dataset from qualified researchers trained in human subject confidentiality protocols may be sent to Dr Siana Jones at the MRC Unit for Lifelong Health and Aging at UCL (siana.jones@ucl.ac.uk).
